# AFFECTING COGNITION: HOW DO POSITIVE AND NEGATIVE AFFECT PREDICT THE FUTURE COGNITIVE PERFORMANCE OF MIDLIFE ADULTS

**DOI:** 10.1093/geroni/igz038.2428

**Published:** 2019-11-08

**Authors:** Jungmin Lee, Sara B W Troutman, Victoria Gertel, Peter Molenaar, David M Almeida

**Affiliations:** 1 The Pennsylvania State University, State College, Pennsylvania, United States; 2 The Pennsylvania State University, The Pennsylvania State University, United States; 3 The Pennsylvania State University, University Park, Pennsylvania, United States

## Abstract

The purpose of the present study is to examine whether positive affect and negative affect can predict the changes in cognitive abilities, assessed by episodic memory and executive functioning, during the transition to late adulthood. Using the longitudinal data from the national survey of midlife development in the United States (MIDUS), this study implements a cross-lagged panel model to test the significance of two pathways; one from affect to cognition and the other in the opposite direction. The results show the three main findings: First, the relationship between affect and cognition is unidirectional in which only positive affect and negative affect during the first occasion significantly predict episodic memory in the second occasion. Second, regardless of the type of affect, higher levels of both positive and negative affect predicts worse episodic memory and this tendency is more prominent in older adults. The combined results emphasize the benefits of using affect as a predictor of changes in the specific types of cognition and suggest using the longitudinal model to account for complex relationships between these psychological constructs.

